# Using deep learning to quantify the beauty of outdoor places

**DOI:** 10.1098/rsos.170170

**Published:** 2017-07-19

**Authors:** Chanuki Illushka Seresinhe, Tobias Preis, Helen Susannah Moat

**Affiliations:** 1 Data Science Lab, Behavioural Science, Warwick Business School, University of Warwick, Coventry CV4 7AL, UK; 2 The Alan Turing Institute, British Library, 96 Euston Road, London NW1 2DB, UK

**Keywords:** environmental aesthetics, well-being, convolutional neural networks, deep learning, online data

## Abstract

Beautiful outdoor locations are protected by governments and have recently been shown to be associated with better health. But what makes an outdoor space beautiful? Does a beautiful outdoor location differ from an outdoor location that is simply natural? Here, we explore whether ratings of over 200 000 images of Great Britain from the online game *Scenic-Or-Not*, combined with hundreds of image features extracted using the Places Convolutional Neural Network, might help us understand what beautiful outdoor spaces are composed of. We discover that, as well as natural features such as ‘Coast’, ‘Mountain’ and ‘Canal Natural’, man-made structures such as ‘Tower’, ‘Castle’ and ‘Viaduct’ lead to places being considered more scenic. Importantly, while scenes containing ‘Trees’ tend to rate highly, places containing more bland natural green features such as ‘Grass’ and ‘Athletic Fields’ are considered less scenic. We also find that a neural network can be trained to automatically identify scenic places, and that this network highlights both natural and built locations. Our findings demonstrate how online data combined with neural networks can provide a deeper understanding of what environments we might find beautiful and offer quantitative insights for policymakers charged with design and protection of our built and natural environments.

## 1. Background

Governments around the world spend a great deal of money preserving and creating beautiful places [1]. As individuals, we often seek such locations out when desiring rest and relaxation. However, the beauty of outdoor spaces has long been considered an intangible measure that is difficult to quantify due to its subjective nature. Outdoor beauty is often considered synonymous with ‘nature’, as evidenced by the major efforts taken to preserve areas in the countryside [2] such as Outstanding Areas of Natural Beauty, and the plethora of landscape paintings presented in museums. Yet, should we deem all natural areas worthy of protection? What about areas that are not primarily natural? What environments in towns and cities might also be considered beautiful, and thus worthy of preservation? If we can quantify the beauty of outdoor spaces, we can find answers to such questions.

While individual ideas of beauty are likely to be shaped by our personal cultural and social experiences, there is also reason to believe that our preferences for certain environments are shaped by evolution [3–5]. Such preferences may not only be for natural elements [6,7], but also for areas with wide vantage points [3], moderate levels of complexity [8–10] and enclosedness [11]. Thus, it is feasible to suppose that there is a collective sense of beauty that we can measure, and that this may not in fact coincide wholly with only natural beauty.

Traditionally, small-scale surveys have been the most cost-effective method of gathering quantifiable data on what people find beautiful in outdoor spaces. Such surveys have provided important initial evidence that beautiful spaces may encourage physical activity [12,13]. However, small-scale surveys have limited scope in terms of which characteristics of environments they can explore, and have generally only explored a handful of characteristics at a time, such as the presence of natural elements [14–16], fractal elements [17,18] or complexity [8–10].

The ability to crowdsource large amounts of data, coupled with recent advances in computer vision methods, is opening up a new avenue for research, allowing us to investigate visual perceptions of our environment. A recent analysis of over 1.5 million ratings of over 200 000 outdoor images taken across Great Britain, crowdsourced via the online game *Scenic-Or-Not,* provided evidence that people who live in more scenic environments report their health to be better [19]. Crowdsourcing has also been used to collect large databases of human perceptions of city images such as ‘safety’, ‘beauty’ and ‘happiness’ [20,21]. Computer vision methods such as ‘sparse coding’ [22] and ‘bag of visual words’ [23] have allowed researchers to identify statistical characteristics and specific areas of images that relate to concepts such as ‘artistic style’ [24] or visual perceptions of cities [25]. More recently, the introduction of convolutional neural networks (CNNs) has led to dramatic improvements in computer vision tasks, including visual recognition [26,27], understanding image aesthetics [28,29] and extracting perceptions of urban neighbourhoods [30,31].

We draw on this ongoing and rapid improvement in computer vision, particularly with CNNs. We use the Places CNN [32,33] to extract hundreds of features from over 200 000 outdoor images from across Great Britain, rated via the online game *Scenic-Or-Not*, in order to develop a deeper and broader understanding of what beautiful outdoor spaces are composed of. We attempt to find answers to our question that go beyond the simple explanation ‘what is natural is beautiful’. Finally, we evaluate to what level of accuracy we can create a model to predict the beauty of scenes for which we do not have survey or crowdsourced scenicness data.

## 2. Exploring the composition of beautiful outdoor scenes

We explore data extracted from images from *Scenic-Or-Not*, an online game that crowdsources ratings of the scenicness of outdoor images. *Scenic-Or-Not* presents users with random geotagged photographs of Great Britain, which visitors can rate on an integer scale 1–10, where 10 indicates ‘very scenic’ and 1 indicates ‘not scenic’. Each image represents a 1 km grid square of Great Britain, and is sourced from *Geograph* (http://www.geograph.org.uk/), an online documentation project encouraging users to submit geographically representative photographs of Great Britain. The *Scenic-Or-Not* dataset comprises 217 000 images covering nearly 95% of the 1 km grid squares of Great Britain. To date, over 1.5 million ratings have been submitted. We only include images in our analysis that have been rated more than three times.

Ratings from *Scenic-Or-Not* have previously been used to explore the links between scenicness and land cover [35], and scenicness and health [19]. Previous research with this data has also investigated whether data from the photo-sharing website Flickr can be used to estimate scenicness [34]. In this study, we use the *Scenic-Or-Not* dataset to understand what characteristics beautiful images of our environment might be composed of. For each *Scenic-Or-Not* image, we use the Places205 AlexNet CNN [32] that has been trained on data from the Scene UNderstanding (SUN) attribute database [36] to extract the probabilities of 102 scene attributes such as ‘trees’ and ‘flowers’. The SUN attribute database contains 102 discriminative outdoor scene attributes, spanning from materials to activities (e.g. ‘wire’, ‘vegetation’, ‘shopping’). We extract probabilities for scene attributes from the FC7 layer (the penultimate fully connected layer) of the AlexNet CNN. Table S1 in the electronic supplementary material lists all the scene attributes used in our analysis.

We use the more recent Places365 CNN trained on the Places2 dataset (a repository of 8 million scene photographs) [33] to extract the probabilities of 365 place category classifications such as ‘mountain’, ‘lake natural’, ‘residential neighbourhood’ and ‘train station platform’. We specifically use the Places365 trained using the 152-layer Residual Network (ResNet152) architecture [37], as this resulted in the best classification accuracy. Table S2 in the electronic supplementary material lists all place categories used in our analysis.

We also explore the basic characteristics of photographs in our scenic ratings dataset, including their colour composition, saturation, brightness and colour variations. We examine each image from *Scenic-Or-Not* on a per-pixel level, with each pixel being allocated to one of 11 colours that constitute the principal colours in the English vocabulary (black, blue, brown, grey, green, orange, pink, purple, red, white and yellow). More details of this procedure and the empirical data that support it can be found in the electronic supplementary material.

**Figure 1.**
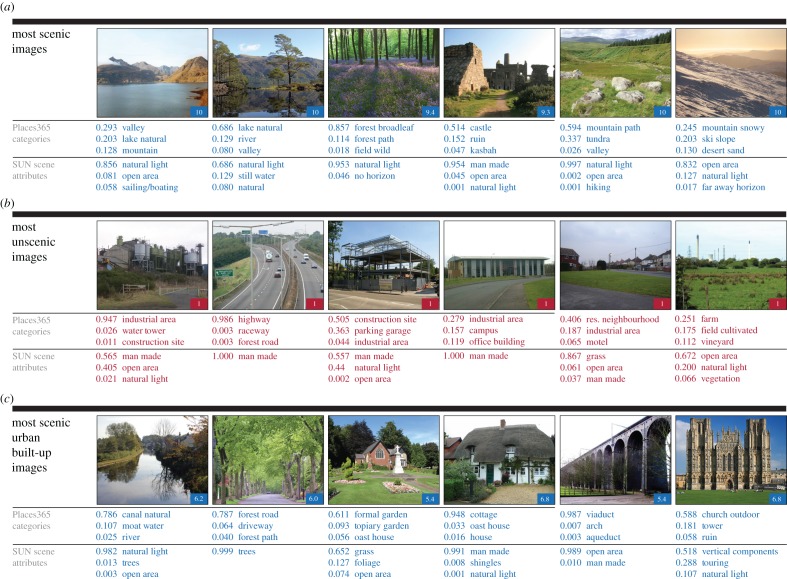
(*Opposite.*) Top three place categories and top three scene attributes of sample scenic and unscenic images across Great Britain. To help us understand what elements comprise scenic and unscenic images, for each *Scenic-Or-Not* image, we extract the probability of 102 scene attributes (e.g. ‘natural’, ‘man made’ and ‘open area’) and 365 place categories (e.g. ‘mountain’, ‘lake natural’, ‘residential neighbourhood’) using the Places CNN [30,31]. Note that only those categories and features given a probability of 0.001 or higher have been included in the figure. (*a*) A sample of the top 5% scenic images seem to accord with widespread notions of beautiful scenery and are composed of rugged mountains, picturesque lakes, lush forests, abundant greenery, charming ruins and scenes where one can view the distant horizon. (*b*) Unscenic images appear to be mainly composed of man-made features, e.g. industrial areas, road networks, construction sites and unsightly buildings. However, we also find images composed of large natural areas scoring as unscenic, such as large areas of bland grass, or beautiful fields hindered by unsightly industrial elements in the distance. (*c*) We specifically look at images that are in urban areas, and are specifically in a built-up rather than natural area, which are often associated with beautiful scenery. A sample of the top 5% of scenic images reveals that some scenic images in urban built-up areas are reminiscent of countryside scenery, including water features and trees. However, the most scenic images in urban built-up areas can also include man-made features such as gardens, bridges or historical architecture. Owing to the different shapes of the photographs, some images have been cropped to aid presentation in this figure. Full URLs for the original images are provided in the electronic supplementary material. Photographers of scenic images: © Gordon Hatton, © Jerry Sharp, © Andrew Smith, © Chris Allen, © Peter Standing, © Richard Webb. Photographers of unscenic images: © Oliver Dixon, © Mat Fascione, © Jeff Tomlinson, © Gordon Brown, © Graham Clutton, © Mike Harris. Photographers of scenic urban built-up images: © David Pinney, © N Chadwick, © David Roberts, © Jonathan Billinger, © John Salmon, © Mike Searle. Copyright of the images is retained by the photographers. Images are licensed for reuse under the Creative Commons Attribution-Share Alike 2.0 Generic License. To view a copy of this licence, visit http://creativecommons.org/licenses/by-sa/2.0/.

Visual inspection of a sample of the most highly scenic images suggests that they conform to widely held notions of beautiful scenery, comprising rugged mountains, bodies of water, abundant greenery and sweeping views (figure 1*a*). The sample of least scenic images suggests that such images are often composed of primarily man-made objects such as industrial areas and highways. However, images containing large areas of natural greenery can also be considered unscenic if they look drab, or if man-made objects, such as industrial plants, are obstructing the view (figure 1*b*).

We also look at a subset of images that are located in urban areas and do not consist primarily of natural land cover that might be associated with beautiful scenery. We differentiate urban areas from rural areas using area classification data from national statistics sources [38,39]. We use data on land cover from the *25 m-resolution UK Land Cover Map 2007 (LCM)* [40] to identify images that are located in primarily built-up rather than natural areas. Table S3 in the electronic supplementary material lists which land cover types have been deemed natural versus built-up.

The sample of images we inspect suggests that the definition of scenicness in urban built-up settings is more varied than in rural areas (figure 1*c*). It appears that the most scenic images in urban areas consist not only of images that might be reminiscent of countryside scenery—such as beautiful canals and tree-lined paths—but of images that also contain man-made features such as historical architecture and bridge-like structures.

The number of photographs in our dataset vastly exceeds a number that could be reasonably examined and characterized by a human encoder. In order to exploit the information contained in all of the photographs in our dataset, rather than a small sample, we build an elastic net model that considers the following features we have extracted from the images: colour composition, 102 SUN scene attributes and those Places365 place categories that are labelled as outdoor categories, of which there are 205. (Note that these 205 outdoor categories from the Places365 CNN differ from the 205 outdoor and indoor categories from the Places205 CNN.) We specifically choose to use an elastic net model as they have been shown to perform well even in situations where there are highly correlated predictors [41]. Elastic net models are a compromise between ridge regression and LASSO (Least Absolute Shrinkage and Selection Operator), both of which are adaptations of the linear regression model, with a penalty parameter in order to avoid overfitting. We use cross-validation to learn the alpha parameter of the elastic net (the mix between ridge and lasso) as well as the lambda parameter (the penalty).

**Figure 2.**
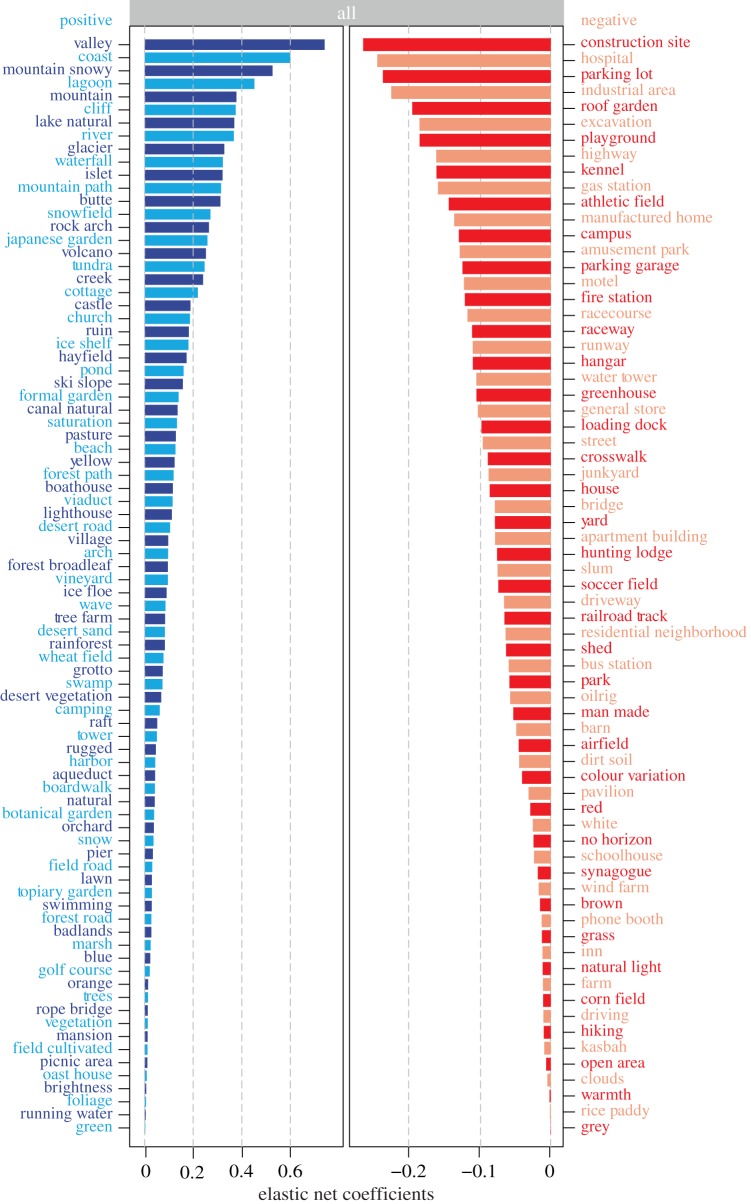
(*Opposite.*) Elastic net coefficients for all areas in Great Britain. We build an elastic net model to identify features that might be most relevant for understanding scenicness. We include features related to the colour composition of images such as the percentage of a selection of 11 colours, as well as ‘saturation’ and ‘brightness’ and ‘colour variation’. We also include 102 scene attributes (e.g. ‘natural’, ‘man made’ and ‘open area’) and 205 outdoor place categories (e.g. ‘mountain’, ‘lake natural’, ‘residential neighbourhood’), which have been extracted using the Places CNN [30,31]. Tables S1 and S2 in the electronic supplementary material list all the scene attributes and the outdoor place categories that were included in the model. The model accords with intuition, whereby natural features are most associated with greater scenicness, such as ‘Valley’, ‘Coast’ and ‘Mountain’, while man-made features such as ‘Construction Site’ and ‘Industrial Area’ are most associated with lower scenicness. However, man-made features such as ‘Cottage’, ‘Castle’ and ‘Lighthouse’ are also associated with greater scenicness. In line with Appleton's prospect–refuge theory [3], we also see features depicted in the results such as ‘No Horizon’ and ‘Open Areas’, which might reflect preferences shaped by our evolution. We examine this further in the Discussion. Note that the *x*-axes for the positive and negative coefficients have different scales.

**Figure 3.**
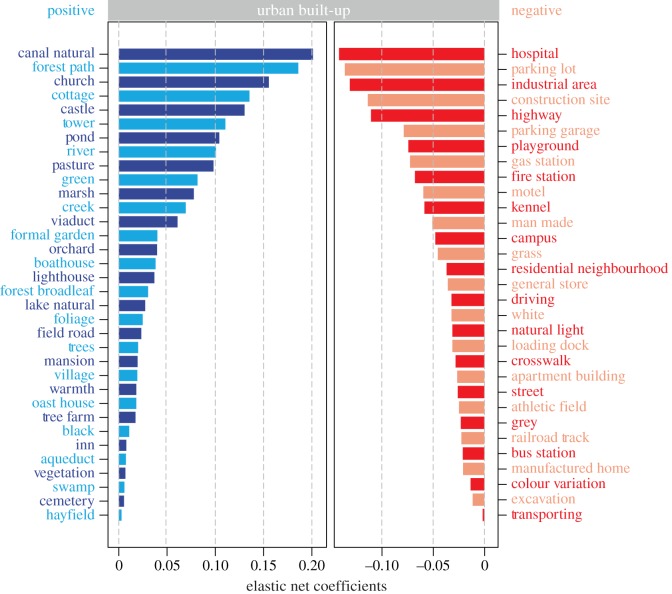
Elastic net coefficients for urban built-up areas in Great Britain. We build an elastic net model to identify features that might be most relevant for understanding scenicness in built-up urban areas, which might have their own definition of scenicness. We include features related to the colour composition of images such as the percentage of a selection of 11 colours, as well as ‘saturation’ and ‘brightness’ and ‘colour variation’. We also include 102 scene attributes (e.g. ‘natural’, ‘man made’ and ‘open area’) which have been extracted using the Places205 CNN [32] and 205 outdoor place categories (e.g. ‘mountain’, ‘lake natural’, ‘residential neighbourhood’) which have been extracted using the Places365 CNN [33]. Tables S1 and S2 in the electronic supplementary material list all the scene attributes and the outdoor place categories that were included in the model. We do indeed find that the definition of scenicness is different for urban built-up locations. We see that natural features that one might more commonly encounter in urban settings such as ‘Canal Natural’, ‘Pond’ and ‘Trees’ are most associated with greater scenicness. We also see historical buildings such as ‘Church’, ‘Castle’ and ‘Tower’, as well as bridge-like structures such as ‘Aqueduct’ are associated with greater scenicness. Interestingly, in both the model trained on urban built-up areas (depicted here) and the model trained on all of our *Scenic-Or-Not* images (depicted in figure 2), large flat areas of greenspace such as ‘Grass’ and ‘Athletic Field’ are associated with lower scenicness. Note that the *x*-axes for the positive and negative coefficients have different scales.

Figures 2 and 3 present the features that the elastic net model determines lead to higher and lower scenic ratings, both across the dataset as a whole, and within urban built-up settings in particular. The model accords with intuition, where natural features are most associated with greater scenicness. These include ‘Valley’, ‘Coast’ and ‘Mountain’ for the full dataset (figure 2) and ‘Canal Natural’, ‘Pond’, ‘Gardens’ and ‘Trees’ in urban built-up settings (figure 3). Man-made features such as ‘Construction Site’, ‘Industrial Area’, ‘Hospital’, ‘Parking Lot’ and ‘Highway’ are most associated with lower scenicness in both models. Interestingly, however, we also see feature associations that contradict the ‘what is natural is beautiful’ explanation. In both models, man-made elements can also lead to higher scenic ratings, including historical architecture such as ‘Church’, ‘Castle’, ‘Tower’ and ‘Cottage’, as well as bridge-like structures such as ‘Viaduct’ and ‘Aqueduct’. Large areas of greenspace such as ‘Grass’ and ‘Athletic Field’ appear to be unscenic in both models. We hypothesize that this might be due to the fact that images composed primarily of flat grass may lack other scenic features such as trees or hills. We also see features that might have been shaped by our evolved preferences coming out in the results. ‘No Horizon’ and ‘Open Area’ are both negatively associated with scenicness in our model containing all images (figure 2).

**Figure 4.**
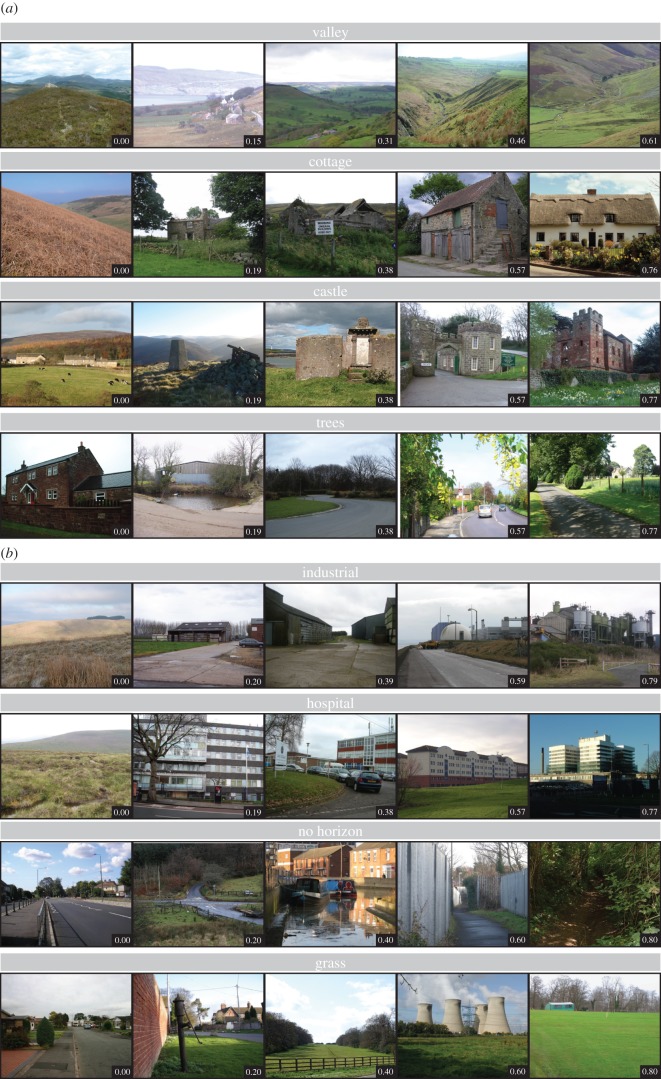
(*Opposite.*) Sample images of features extracted via the Places CNN. For each image, we extract scene attributes and place categories using the Places CNN [30,31], which assigns a probability score to each attribute. For each attribute, we split the range of probabilities into five equal intervals, and extract a sample image from each interval. (*a*) Sample images with features that are most positively associated with scenicness. Natural features, such as ‘Valley’ and ‘Trees’, are understandably associated with more scenicness. However, we also find that certain types of man-made structures, such as ‘Castle’ and ‘Viaduct’, are positively associated with scenicness. (*b*) Sample images with features that are most negatively associated with scenicness. As expected, images that are primarily ‘Industrial’ or contain unsightly man-made objects are not considered as scenic as those without such features. We also find that if a scene has a restricted field of view, such ‘No Horizon’, images are also rated as unscenic. Surprisingly, we find ‘Grass’ is also negatively associated with scenicness. It might be that images that contain the most grass lack other features such as trees or hill contours, resulting in an uninteresting scene. Owing to the different shapes of the photographs, some images have been cropped to aid presentation in this figure. Full URLs for the original images are provided in the electronic supplementary material. Photographers of ‘Valley’ images: © Alan Stewart, © Anne Burgess, © Joe Regan, © Chris Wimbush, © Chris Eilbeck. Photographers of ‘Trees’ images: © Alexander P Kapp, © Bob Jenkins, © Tom Pennington, © Colin Smith, © James Allan. Photographers of ‘Castle’ images: © Gordon Hatton, © Iain Macaulay, © Anne Burgess, © David Smith, © Ceri Thomas. Photographers of ‘Cottage’ images: © Eirian Evans, © Dennis Thorley, © Jeff Collins, © Colin Grice, © Robert Edwards. Photographers of ‘Industrial’ images: © John Lucas, © Jonathan Billinger, © Chris Heaton, © M J Richardson, © Oliver Dixon. Photographers of ‘Hospital’ images: © Richard Webb, © Chris L L, © Colin Bates, © Iain Thompson, © Robin Hall. Photographers of ‘No Horizon’ images: © Dr Neil Clifton, © Nigel Brown, © Kate Nicol, © Row17, © Oliver Dixon. Photographers of ‘Grass’ images: © Stephen Pearce, © Row17, © Rob Farrow, © Paul Glazzard, © Mike Quinn. Copyright of the images is retained by the photographers. Images are licensed for reuse under the Creative Commons Attribution-Share Alike 2.0 Generic License. To view a copy of this licence, visit http://creativecommons.org/licenses/by-sa/2.0/.

Figure 4 shows sample images from some of the features mentioned above. Indeed, we can clearly see that large areas of ‘Grass’ might be rated as unscenic as they might lack interesting characteristics such as the contours found in ‘Valley’. The images with ‘No Horizon’ appear to be those that lack a clear view of the surroundings.

## 3. Predicting scenicness

We now check to what degree we can predict the beauty of scenes for new places for which we do not have crowdsourced scenicness data. We first build an elastic net model to predict the scenicness of images. This time we hold out 20% of our data to test our prediction accuracy. Our performance measure is the Kendall's rank correlation between the predicted scenic scores and the actual scenic scores. With our model applied to all images, we achieve a performance score of 0.544 for all images and 0.445 for our urban built-up images.

As CNNs have shown tremendous progress in computer vision tasks [26–31], we also investigate whether scenic ratings can be directly predicted by a customized CNN. Previous work has investigated whether CNNs can be used to identify photographs of high aesthetic quality [28,29]. By contrast, here we wish to train a CNN to evaluate the aesthetics of the environment, rather than that of the photograph itself. Note that these two qualities are not identical: e.g. badly composed photographs of beautiful areas may still be recognized as highly scenic, but might not score high in terms of photographic aesthetics.

**Figure 5.**
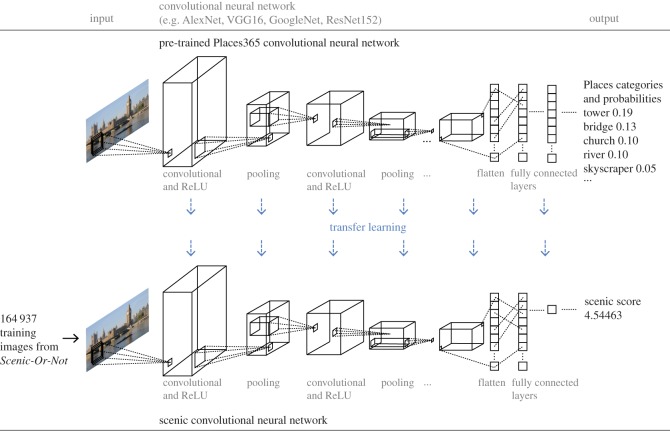
Using transfer learning to predict scenicness. As CNNs have shown tremendous progress in computer vision tasks [40,41], we check whether we can use a CNN to predict the scenic ratings of images with a high degree of accuracy. Here, we provide an abstract illustration of the CNN architecture and our approach. As we have limited training data, we use a transfer learning approach [42] to leverage the knowledge of the Places365 CNN. We modify the final layer of our CNN to predict scenic scores rather than the probabilities of place categories. We fine-tune all the layers of the CNN, trained on the Places365 database, to predict the scenicness of images using the four different CNN architectures that have been used to train the Places365 CNN: AlexNet [43], Visual Geometry Group (VGG16) [44], GoogleNet [45] and ResNet152 [37]. Image © Philip Halling. Copyright of the image is retained by the photographer. Images are licensed for reuse under the Creative Commons Attribution-Share Alike 2.0 Generic License. To view a copy of this licence, visit http://creativecommons.org/licenses/by-sa/2.0/. Figure adapted from Mathworks CNNs webpage figure at https://uk.mathworks.com/discovery/convolutional-neural-network.html.

As we have limited training data, we use a transfer learning approach [42] to leverage the knowledge of the pre-trained Places365 CNN, as this CNN already performs well in scene recognition. Figure 5 illustrates the method used for this approach. We fine-tune all the layers of the CNN, trained on the Places365 database, to predict the scenicness of images. We examine the performance of all four different CNN architectures that have been used to train the Places365 CNN: AlexNet [43], Visual Geometry Group (VGG16) [44], GoogleNet [45] and ResNet152 [37]. For all our experiments, we use the deep learning framework Caffe [46]. For AlexNet, VGG16 and GoogleNet, training is performed by stochastic gradient descent (SGD) with mini-batch size 50, a learning rate 0.0001 and momentum 0.9 for 10 000 iterations. For ResNet152, training is performed using a mini-batch size of 10 (due to GPU memory constraints) for 50 000 iterations, to ensure all four networks were exposed to the same amount of images.

Table 1 compares the results for both the elastic net and all the fine-tuned CNN models. The Scenic CNN trained using the VGG16 CNN architecture delivers the best performance for all images, achieving a performance score of 0.658 for all images and 0.590 for our urban built-up images, measured again using Kendall's rank correlation. The performance of the slightly deeper GoogleNet and the much deeper ResNet152 models are similar. Further experiments could be carried out in the future to determine if the deeper networks can be made to perform better, perhaps by varying training parameters (e.g. by choosing different learning rates or different optimization techniques). However, it might be the case that for this task, the deeper networks may be more prone to overfitting, and thus may not generalize well [47]. Further experiments would be required to conclusively state which network might be best suited for prediction of scene aesthetics.

**Table 1.** Scenic prediction results. We check to what degree we can predict the beauty of scenes for new places for which we do not have survey or crowdsourced scenicness data. Our first model is an elastic net model to predict the scenicness of images. Our second model is a CNN fine-tuned on the Places365 CNN to predict the scenicness of images. We check the performance on four different CNN architectures that have been used to train the Places365CNN: AlexNet [43], Visual Geometry Group (VGG16) [44], GoogleNet [45] and ResNet152 [37]. We hold out a 20% test set to check our prediction accuracy. We calculate a performance measure using the Kendall rank correlation between the predicted scenic scores and the actual scenic scores. All four Scenic CNNs outperform the elastic net model in both of our datasets, with all *Scenic-Or-Not* images, and also with only Urban Built-up *Scenic-Or-Not* images. The Scenic CNN trained using the VGG16 CNN architecture delivers the best performance overall.

		scenic CNN			
	elastic net	AlexNet	VGG16	GoogleNet	ResNet152
all	0.544	0.627	0.658	0.653	0.654
urban built-up	0.445	0.553	0.590	0.590	0.567

**Figure 6.**
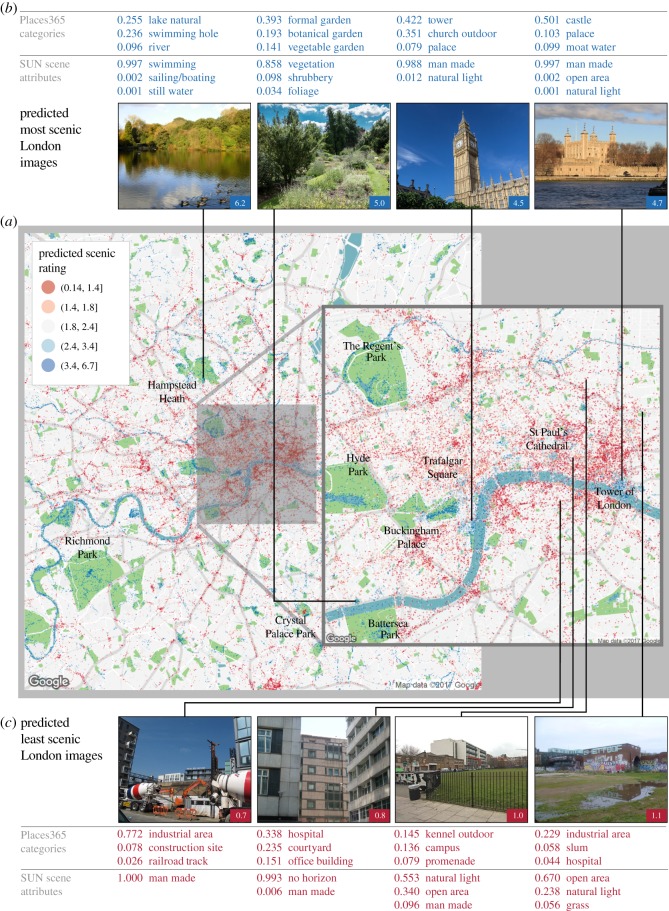
(*Opposite.*) Predictions of scenic ratings for London images. In order to predict the scenic ratings of images for which we do not already have crowdsourced data, we use a transfer learning approach to leverage the knowledge of the Places365 CNN [24], which can predict the place category of a scene with a high degree of accuracy. We modify the Places CNN to instead predict the scenicness of an image. We check the performance on four different CNN architectures that have been used to train the Places365CNN: AlexNet [43], Visual Geometry Group (VGG16) [44], GoogleNet [45] and ResNet152 [37]. We hold out a 20% test set to check our prediction accuracy. We calculate a performance measure using the Kendall rank correlation between the predicted scenic scores and the actual scenic scores. The Scenic CNN trained using the VGG16 CNN architecture delivers the best performance with an overall prediction accuracy of 0.658. With our new Scenic CNN, we predict the scenicness of pictures of London uploaded to *Geograph* (http://www.geograph.org.uk/), an online project that collects geographically representative photographs of Great Britain and Ireland. Note that only those categories and features given a probability of 0.001 or higher have been included in the figure. (*a*) Examining the estimates of how scenic images around London are, we immediately notice that parks known for their stunning scenery such as Hampstead Heath and Richmond Park have large clusters of images rated as scenic. The city centre appears to be largely unscenic, although a close-up view reveals clusters of scenic images in built-up areas. (*b*) A sample of the top 5% of the photos predicted as scenic indicates that our Scenic CNN mostly predicts high ratings for images containing primarily natural elements. However, we also see that images containing primarily man-made objects can also be estimated as scenic. Notably, our Scenic CNN has also picked two well-known icons of London—Big Ben and the Tower of London—and rated them as scenic. This is in line with the results of our elastic net analysis, where ‘Tower’ and ‘Castle’ are features that are significantly associated with scenicness. (c) A sample of the bottom 5% of the photos predicted as scenic indicates that our CNN predicts low ratings for images containing primarily man-made features. Images with a restricted view can also be rated as scenic. However, images containing large areas of greenspace also tend to be rated low if they are largely flat and uninteresting or unmaintained. Owing to the different shapes of the photographs, some images have been cropped to aid presentation in this figure. Full URLs for the original images are provided in the electronic supplementary material. Photographers of scenic images: © Stephen McKay, © Christine Matthews, © Christine Matthews, © Roger Davies; Photographers of unscenic images: © Stephen Craven, © Robert Lamb, © John Salmon, © Marathon. Copyright of the images is retained by the photographers. Images are licensed for reuse under the Creative Commons Attribution-Share Alike 2.0 Generic License. To view a copy of this licence, visit http://creativecommons.org/licenses/by-sa/2.0/.

Our *Scenic-Or-Not* database contains only one image per 1 km^2^ grid square, and only in Great Britain. We check how well our Scenic CNN performs in an area where we do not have images at a high resolution from *Scenic-Or-Not*. Specifically, we investigate how our Scenic CNN performs for London by predicting the scenic ratings of 243 339 outdoor London images uploaded to *Geograph.* We use the Places CNN [32] to determine whether an image has been taken outdoors. The labels of the top five predicted place categories can be used to check if the given image is indoors or outdoors with more than 95% accuracy [32]. With a performance accuracy of 0.658, we find that, in general, our scenic estimates from the CNN accord with what we might expect. Figure 6*a* demonstrates that parks known for their scenery, such as Hampstead Heath and Richmond Park, have large clusters of scenic imagery. We also see that areas around large bodies of water such as the Thames also seem to contain the most scenic imagery. The most unscenic images seem to be located in the city centre. However, a close-up view reveals clusters of highly scenic imagery in attractive built-up areas, such as Trafalgar Square. An examination of the photos predicted to be scenic indicates that while our Scenic CNN predicts high ratings for images containing primarily natural elements, images of man-made elements, particularly historical architecture around the city, including Big Ben and the Tower of London, are also predicted to be scenic (figure 6*b*). While our Scenic CNN in general predicts low ratings for images containing primarily man-made features, images containing large areas of drab or unmaintained greenspace and images with a restricted view are also rated as unscenic (figure 6*c*).

## 4. Conclusion

We consider whether crowdsourced data generated from over 200 000 images from the existing online game *Scenic-Or-Not*, combined with the ability to extract hundreds of features from the images using the CNN Places365, might help us understand what beautiful outdoor spaces are composed of. We attempt to find answers to our question that go beyond the simple explanation that ‘what is natural is beautiful’, and explore what features contribute to beauty in urban and built-up settings.

We find, as expected, that natural features, such as ‘Coast’ and ‘Mountain’, are indeed associated with greater scenicness. However, in urban built-up areas, the definition of scenicness varies, and instead we see that natural features such as ‘Pond’, ‘Garden’ and ‘Trees’ are associated with greater scenicness. Surprisingly, we also find that man-made features can also be rated as scenic, in general as well as in urban built-up settings specifically. We find that historical buildings, such as ‘Cottage’ and ‘Castle’, as well as bridge-like structures, such as ‘Viaduct’ and ‘Aqueduct’, are associated with greater scenicness.

What we find to be unscenic might provide the greatest insights. While, as expected, we find that man-made features such as ‘Construction Site’ and ‘Parking Lots’ are associated with lower scenicness, large areas of greenspace such as ‘Grass’ and ‘Athletic Field’ can also lead to lower scenic ratings. Evolution might have conditioned us to dislike certain natural settings if they have attributes that are detrimental to our survival [4]. For example, we seem to dislike certain natural settings if they appear to be drab or neglected [48], or simply uninteresting to explore [9,10]. We also find that ‘No Horizon’ and ‘Open Spaces’ are also associated with lower scenicness. This accords with Jay Appleton's theory of ‘prospect and refuge’ [3], which suggests that humans have evolved to prefer outdoor spaces where one can easily survey ‘prospects’ and which contain ‘refuge’ where one can easily hide and avoid potential dangers.

Finally, we also explore to what level of accuracy we can create a model to predict the beauty of scenes for which we either do not have crowdsourced scenic ratings, or for which we require scenic ratings at a higher resolution. We modify the existing Places365 CNN in order to predict the scenicness of images and achieve the best performance using the VGG16 CNN architecture. As well as carrying out a quantitative analysis of the performance of our CNN, we present our predictions for images in London, and find that they are broadly in line with intuition. Our Scenic CNN predicts high ratings for images containing primarily natural elements, such as those located in parks in London known for their attractive scenery, such as Hampstead and Richmond Park, and also predicts high scenic ratings for beautiful buildings, such as the iconic Big Ben and the Tower of London.

In order to improve the prediction performance of our model, we anticipate that further data to differentiate particular features of built-up areas are needed. For example, we note that while we find historical buildings (e.g. ‘Castle’) to be the most beautiful, this could reflect the fact that we do not have categories to describe modern types of architecture in our data. Future research could explore this further.

In general, our findings offer insights which may help inform how we might design spaces to increase human well-being. It appears that the old adage ‘natural is beautiful’ seems to be incomplete: flat and uninteresting green spaces are not necessarily beautiful, while characterful buildings and stunning architectural features can be. Particularly in urban areas, features such as ponds and trees seem to be important for city beauty, while spaces that feel closed-off or those that are too open and offer no refuge seem to be spaces that we do not rate as beautiful and do not prefer to spend time in. This accords with research that investigates whether our preferences for certain environments might be shaped by evolution, which explains our attraction not only to natural spaces [6,7] but also to ones where we might feel more safe [3] or spaces that are interesting to explore [8–10].

Our findings demonstrate that the availability of large crowdsourced datasets, coupled with recent advances in neural networks, can help us develop a deeper understanding of what environments we might find beautiful. Crucially, such advances can help us develop vital evidence necessary for policymakers, urban planners and architects to make decisions about how to design spaces that will most increase the well-being of their inhabitants.

## Supplementary Material

Supplementary Material

## Data accessibility

This study was a re-analysis of existing data that are publicly available. Data on *Scenic-Or-Not* ratings are openly available at http://scenicornot.datasciencelab.co.uk. We retrieved scenicness ratings by accessing the *Scenic-Or-Not* website on 2 August 2014. The *Scenic-Or-Not* dataset used in this study is available via the Dryad Digital Repository (http://dx.doi.org/10.5061/dryad.rq4s3) [49]. *Geograph* images are openly available to download from http://www.geograph.org.uk/. We retrieved images of London from *Geograph* on 25 October 2016. The dataset of *Geograph* images used in figure 6 is available via the Dryad Digital Repository (http://dx.doi.org/10.5061/dryad.rq4s3) [49]. The Places CNNs are openly available to download at http://places.csail.mit.edu/. The Caffe deep learning framework [46] can be accessed at http://caffe.berkeleyvision.org/. The glmnet package in R was used for the elastic net model implementation [48].
