# Supplementary material for: Using deep learning to quantify the beauty of outdoor places
Source: R Soc Open Sci. 2017 Jul 19;4(7):170170. doi: 10.1098/rsos.170170 (PMC5541537; doi:10.1098/rsos.170170)
Supplement: Supplementary Material [file rsos170170supp1.pdf]

TITLE

Using Deep Learning to Quantify the Beauty of Outdoor Places

Supplementary Material

AUTHORS

Chanuki Illushka Seresinhe[1,2*], Tobias Preis[1,2] and Helen Susannah Moat[1,2]

AFFILIATIONS

[1] Data Science Lab, Behavioural Science, Warwick Business School,

University of Warwick, Coventry, CV4 7AL, UK

[2] The Alan Turing Institute, British Library, 96 Euston Road, London, NW1 2DB, UK

* To whom correspondence should be addressed;

E-mail: C.Seresinhe@warwick.ac.uk

SUPPLEMENTARY MATERIAL

Extracting the colour features from images

As colour naming varies from one individual to another [1], we draw on crowdsourced data generated through an online survey of 1.5 million participants [2] to determine to which colour a pixel should be allocated. In this survey, participants were shown an area filled with a random fully-saturated colour on both black and white backgrounds, and asked to name the colour. These responses were then used to create a list of the dominant colour names corresponding to fully saturated RGB (Red, Green, Blue) values. We use this data in order to determine where colour boundaries should be drawn: for example, where "brown" ends and "green" begins. The RGB colours are converted to the HSV (Hue, Saturation, Value) colour space and each pixel is matched to the closest corresponding colour based on its hue parameter. The nature of the relationship between HSV and RGB space is such that all possible hues are covered by all fully saturated RGB colours. As black, grey and white do not have a defined hue, these colour boundaries were determined based on a combination of the levels of "Saturation" and "Value" (Fig. S1).

We measure the saturation of each image by calculating the mean "Saturation" of each pixel in the HSV colour space. We measure the brightness of each image by calculating the mean "Value" of each pixel in the HSV colour space. We measure the colour variation of each image by using k-means clustering to reduce the colour palette of each image to an eight colour palette. We then compute the mean R, G and B values of the colour palette, and then derive a measure for how much colour variation is in the image, by taking the square root of the sum of squares of each palette colour's R, G and B difference from the mean.

URLs for *Geograph* images used in figures

FIGURE 1
Scenic Images
http://www.geograph.org.uk/photo/136324
http://www.geograph.org.uk/photo/332167
http://www.geograph.org.uk/photo/800698
http://www.geograph.org.uk/photo/995741
http://www.geograph.org.uk/photo/211685
http://www.geograph.org.uk/photo/1076266

Unscenic Images
http://www.geograph.org.uk/photo/124387
http://www.geograph.org.uk/photo/559081
http://www.geograph.org.uk/photo/1003384
http://www.geograph.org.uk/photo/129255
http://www.geograph.org.uk/photo/101986
http://www.geograph.org.uk/photo/165609

Scenic Urban Built-up Images
http://www.geograph.org.uk/photo/953146
http://www.geograph.org.uk/photo/170042
http://www.geograph.org.uk/photo/909607
http://www.geograph.org.uk/photo/921115
http://www.geograph.org.uk/photo/345984
http://www.geograph.org.uk/photo/986698

Figure 4
Valley
http://www.geograph.org.uk/photo/478904
http://www.geograph.org.uk/photo/7304
http://www.geograph.org.uk/photo/435147
http://www.geograph.org.uk/photo/168140
http://www.geograph.org.uk/photo/52245

Cottage
http://www.geograph.org.uk/photo/375660
http://www.geograph.org.uk/photo/25229
http://www.geograph.org.uk/photo/1012339
http://www.geograph.org.uk/photo/180919
http://www.geograph.org.uk/photo/152474

Castle
http://www.geograph.org.uk/photo/1043725
http://www.geograph.org.uk/photo/630605
http://www.geograph.org.uk/photo/531069
http://www.geograph.org.uk/photo/144367
http://www.geograph.org.uk/photo/789847

Trees
http://www.geograph.org.uk/photo/173682
http://www.geograph.org.uk/photo/118173
http://www.geograph.org.uk/photo/369021
http://www.geograph.org.uk/photo/785541

http://www.geograph.org.uk/photo/197817

Industrial

http://www.geograph.org.uk/photo/1097286
http://www.geograph.org.uk/photo/283577
http://www.geograph.org.uk/photo/1006574
http://www.geograph.org.uk/photo/1036394
http://www.geograph.org.uk/photo/124387

Hospital
http://www.geograph.org.uk/photo/905089
http://www.geograph.org.uk/photo/1122213
http://www.geograph.org.uk/photo/100961
http://www.geograph.org.uk/photo/96830
http://www.geograph.org.uk/photo/109860

No Horizon
http://www.geograph.org.uk/photo/988217
http://www.geograph.org.uk/photo/1067087
http://www.geograph.org.uk/photo/1129057
http://www.geograph.org.uk/photo/1131812
http://www.geograph.org.uk/photo/137040

Grass
http://www.geograph.org.uk/photo/266053
http://www.geograph.org.uk/photo/711812
http://www.geograph.org.uk/photo/775674
http://www.geograph.org.uk/photo/582042
http://www.geograph.org.uk/photo/629610

Figure 6
London Scenic
http://www.geograph.org.uk/photo/1029150
http://www.geograph.org.uk/photo/5036247
http://www.geograph.org.uk/photo/3513652
http://www.geograph.org.uk/photo/2762703

London Unscenic
http://www.geograph.org.uk/photo/5139678
http://www.geograph.org.uk/photo/4019341
http://www.geograph.org.uk/photo/1169461
http://www.geograph.org.uk/photo/3808137

SI Table 1. ***102 Scene UNderstanding (SUN) Scene Attributes*.** We use the Places205 AlexNet CNN [3] trained on data from the Scene UNderstanding (SUN) attribute database [4] to extract the probabilities of the following 102 scene attributes.

sailing/boating	spectating	tiles	glossy
driving	farming	concrete	matte
biking	constructing	metal	sterile
transporting	shopping	paper	moist
sunbathing	medical	wood	dry
touring	working	vinyl	dirty
hiking	using tools	plastic	rusty
climbing	digging	cloth	warm
camping	business	sand	cold
reading	praying	rocky	natural
studying	fencing	dirt soil	man-made
training	railing	marble	open area
research	wire	glass	semi-enclosed area
diving	railroad	waves	enclosed area
swimming	trees	ocean	far-away horizon
bathing	grass	running water	nohorizon
eating	vegetation	still water	rugged
cleaning	shrubbery	ice	vertical components
socializing	foliage	snow	horizontal components
congregating	leaves	clouds	symmetrical
waiting	flowers	smoke	cluttered
competing	asphalt	fire	scary
sports	pavement	natural light	soothing
exercise	shingles	sunny	stressful
playing	carpet	electric lighting	
gaming	brick	aged	

SI Table 2. ***365 Place Categories.*** We use the more recent Places365 CNN trained on the Places2 dataset (a repository of 8 million scene photographs) [5] to extract the probabilities of the following 365 place category classifications such as "mountain", "lake natural", "residential neighbourhood" and "train station platform". In our elastic net model we only consider those features which have been labelled as outdoor place categories as listed below.

**Outdoor**					
Airfield	Carrousel	Forest Path	Lake Natural	Picnic Area	Stage
Alley	Castle	Forest Road	Landfill	Pier	Street
Amphitheater	Cemetery	Formal Garden	Landing Deck	Playground	Swamp
Amusement Park	Chalet	Fountain	Lawn	Plaza	Swimming Pool
Apartment Building	Church	Garage	Library	Pond	Synagogue
Aqueduct	Cliff	Gas Station	Lighthouse	Porch	Temple Asia
Arch	Coast	Gazebo Exterior	Loading Dock	Promenade	Topiary Garden
Army Base	Construction Site	General Store	Lock Chamber	Racecourse	Tower
Athletic Field	Corn Field	Glacier	Mansion	Raceway	Tree Farm
Badlands	Corral	Golf Course	Manufactured Home	Raft	Tree House
Balcony Exterior	Cottage	Greenhouse	Market	Railroad Track	Trench
Balcony Interior	Courthouse	Grotto	Marsh	Rainforest	Tundra
Bamboo Forest	Courtyard	Hangar	Mausoleum	Residential Neighborhood	Underwater Ocean Deep
Barn	Creek	Harbor	Medina	Restaurant Patio	Valley
Barndoor	Crevasse	Hayfield	Moat Water	Rice Paddy	Vegetable Garden
Baseball Field	Crosswalk	Heliport	Mosque	River	Viaduct
Bazaar	Dam	Highway	Motel	Rock Arch	Village
Beach	Desert Sand	Hospital	Mountain	Roof Garden	Vineyard
Beach House	Desert Vegetation	Hot Spring	Mountain Path	Rope Bridge	Volcano
Beer Garden	Desert Road	Hotel	Mountain Snowy	Ruin	Volleyball Court
Boardwalk	Diner	House	Museum	Runway	Water Park
Boat Deck	Doorway	Hunting Lodge	Oast House	Sandbox	Water Tower
Boathouse	Downtown	Ice Floe	Ocean	Schoolhouse	Waterfall
Botanical Garden	Driveway	Ice Shelf	Office Building	Shed	Watering Hole
Bridge	Embassy	Ice Skating.rink	Oilrig	Shopfront	Wave
Building Facade	Excavation	Iceberg	Orchard	Ski Resort	Wheat Field
Bullring	Farm	Igloo	Pagoda	Ski Slope	Wind Farm
Bus Station.indoor	Field Cultivated	Industrial Area	Palace	Sky	Windmill
Butte	Field Wild	Inn	Park	Skyscraper	Yard
Cabin	Field Road	Islet	Parking Garage	Slum	Zen Garden
Campsite	Fire Escape	Japanese Garden	Parking Lot	Snowfield	
Campus	Fire Station	Junkyard	Pasture	Soccer Field	
Canal Natural	Fishpond	Kasbah	Patio	Stadium Baseball	
Canal Urban	Football Field	Kennel	Pavilion	Stadium Football	
Canyon	Forest Broadleaf	Lagoon	Phone Booth	Stadium Soccer	

Indoor					
Airplane Cabin	Basement	Closet	Galley	Martial Arts Gym	Server Room
Airport Terminal	Basketball Court	Clothing Store	Garage	Mezzanine	Shoe Shop
Alcove	Bathroom	Cockpit	General Store	Movie Theater	Shopping Mall
Amusement Arcade	Bazaar	Coffee Shop	Gift Shop	Museum	Shower
Aquarium	Beauty Salon	Computer Room	Greenhouse	Music Studio	Stable
Arcade	Bedchamber	Conference Center	Gymnasium	Natural History Museum	Stage
Archaelogical Excavation	Bedroom	Conference Room	Hangar	Nursery	Staircase
Archive	Beer Hall	Corridor	Hardware Store	Nursing Home	Storage Room
Arena Hockey	Berth	Delicatessen	Home Office	Office	Subway Station Platform
Arena Performance	Biology Laboratory	Department Store	Home Theater	Office Cubicles	Supermarket
Arena Rodeo	Bookstore	Dining Hall	Hospital Room	Operating Room	Sushi Bar
Art Gallery	Booth	Dining Room	Hotel Room	Orchestra Pit	Swimming Hole
Art School	Bow Window	Discotheque	Ice Cream.parlor	Pantry	Swimming Pool
Art Studio	Bowling Alley	Dorm Room	Ice Skating.rink	Parking Garage	Television Room
Artists Loft	Boxing Ring	Dressing Room	Jacuzzi	Pet Shop	Television Studio
Assembly Line	Burial Chamber	Drugstore	Jail Cell	Pharmacy	Throne Room
Atrium Public	Bus Interior	Elevator Door	Jewelry Shop	Physics Laboratory	Ticket Booth
Attic	Butchers Shop	Elevator Lobby	Kindergarden Classroom	Pizzeria	Toyshop
Auditorium	Cafeteria	Elevator Shaft	Kitchen	Playroom	Train Interior
Auto Factory	Candy Store	Engine Room	Laundromat	Pub	Train Station.platform
Auto Showroom	Car Interior	Entrance Hall	Lecture Room	Reception	Utility Room
Bakery Shop	Catacomb	Escalator	Legislative Chamber	Recreation Room	Veterinarians Office
Ball Pit	Chemistry Lab	Fabric Store	Library	Repair Shop	Waiting Room
Ballroom	Childs Room	Fastfood Restaurant	Living Room	Restaurant	Wet Bar
Bank Vault	Church	Flea Market	Lobby	Restaurant Kitchen	Youth Hostel
Banquet Hall	Classroom	Florist Shop	Locker Room	Sauna	
Bar	Clean Room	Food Court	Market	Science Museum	

SI Table 3. ***Land Cover Map Data.*** In order to identify images taken in natural locations, we use data on land cover type from the *25m-resolution UK Land Cover Map 2007 (LCM) [6].* The table shows which land cover types have been deemed as natural, and which have been deemed as not-natural.

**LCM2007 class**	**Habitat**
Broadleaved woodland	Natural
Coniferous woodland	
Arable and Horticulture	
Improved Grassland	
Rough Grassland	
Neutral Grassland	
Calcareous Grassland	
Acid Grassland	
Fen, Marsh and Swamp	
Heather	
Heather grassland	
Bog	
Montane habitats	
Inland Rock	
Salt water	
Freshwater	
Supra-littoral Rock	
Supra-littoral Sediment	
Littoral Rock	
Littoral Sediment	
Saltmarsh	
Urban (including Bare and Urban)	Not Natural
Suburban (including Urban industrial and Urban suburban)	

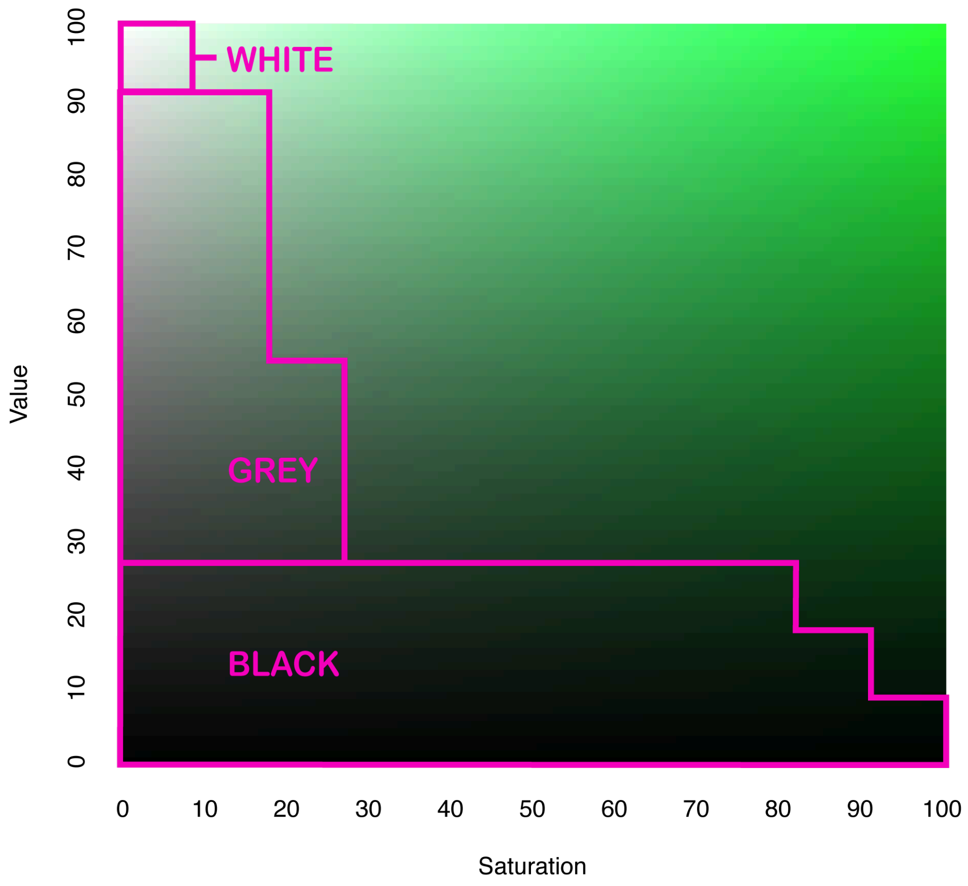

Figure S1 | Allocating black, grey and white based on value and saturation. Here, the hue has been set to one color (green) in order to illustrate where the boundaries of black, grey and white are set. At the borders, the color may appear to be more or less grey, as well as more or less black, depending on the hue, so the boundaries are chosen as a best-fit compromise over the entire range of hue values.

SUPPLEMENTARY MATERIAL REFERENCES

1. Ratliff, F. 1976 On the Psychophysiological Bases of Universal Color Terms. *Proc. Am. Philos. Soc.* **120**, 311-330.
2. XKCD. 2014 *Colour Survey Results.* Available at: https://blog.xkcd.com/2010/05/03/color-survey-results/ (Accessed May 23, 2014).
3. Zhou, B., Lapedriza, A., Xiao, J., Torralba, A., & Oliva, A. 2014 Learning deep features for scene recognition using places database. In *Advances in Neural Information Processing Systems 27 Montreal, Canada, 8 - 13 December 2014,* pp. 487-495.
4. Patterson, G., Xu, C., Su, H., & Hays, J. 2014 The SUN Attribute Database: Beyond Categories for Deeper Scene Understanding. *Int. J. Comput. Vision.* **108**, 59-81 (DOI 10.1007/s11263-013-0695-z).
5. Zhou, B., Khosla, A., Lapedriza, A., Torralba, A., & Oliva, A. 2016 Places: An image database for deep scene understanding. *arXiv preprint arXiv:1610.02055*.
6. Morton, D., Rowland, C., Wood, C., Meek, L., Marston, C., Smith, G., Wadsworth, R., & Simpson, I. 2014 Land cover map 2007 (vector, GB) v1.2. NERC environmental information data Centre. (DOI 10.5285/2ab0b6d8-6558-46cf-9cf0-1e46b3587f13).
